# The Effect of Cement Thickness on Shear Bond Strength of Zirconia with Three Different Self-Adhesive Resin Cements

**DOI:** 10.1055/s-0045-1806933

**Published:** 2025-05-01

**Authors:** Thofun Ieamsuwantada, Suparaksa Yamockul, Niyom Thamrongananskul, Atikom Surintanasarn, Awiruth Klaisiri

**Affiliations:** 1 Department of Prosthodontics, Faculty of Dentistry, Chulalongkorn University, Pathumwan, Bangkok, Thailand; 2 Division of Restorative Dentistry, Faculty of Dentistry, Thammasat University, Pathum Thani, Thailand

**Keywords:** zirconia, self-adhesive resin cement, cement thickness

## Abstract

**Objectives:**

This study aimed to evaluate the influence of resin cement thickness on the shear bond strength (SBS) of zirconia bonded with three different self-adhesive resin cements (SACs).

**Materials and Methods:**

One hundred and twenty zirconia specimens were prepared. Zirconia disks were milled into a cuboidal shape of size 8 × 8 × 6 mm ^3^ using a milling machine, sintered according to the manufacturer's instruction, polished with sandpaper, airborne particle abraded with 50 μm aluminum oxide particles, and randomly divided into 12 groups ( *n* = 10), according to the cement thickness (50, 100, 150, and 200 μm) and SAC (RelyX U200, PANAVIA SA LUTING Multi, and Maxcem Elite Chroma). The specimens were cemented to the resin composite blocks with SAC, stored in water at 37°C for 24 hours, and subjected to the SBS test. Mode of failure was evaluated under a stereo microscope. The data were statistically analyzed using two-way ANOVA and the Tukey HSD test to compare the differences in mean SBSs between groups at 95% confidence level.

**Results:**

Statistical analysis revealed significant differences in SBS ( *p* < 0.05) across the SACs, with the highest mean observed in the 50 μm cement thickness group and decreased as cement thickness increased. PANAVIA SA LUTING Multi exhibited the highest SBS, followed by the RelyX U200 groups, with the lowest SBS observed in Maxcem Elite Chroma groups ( *p* < 0.05). In addition, the predominant mode of failure in all groups, except RelyX U200 and Maxcem Elite Chroma groups with the thickness of 200 μm, was score 4 (no resin cement remained on the zirconia surface).

**Conclusion:**

The difference in cement thickness and the type of SAC affected the SBS to zirconia. The lesser the cement thickness, the higher the SBS. 10-MDP-based cements, which provide the highest bond strength to zirconia and low cement thickness, are suggested for zirconia cementation to achieve a strong bond between zirconia and resin cement.

## Introduction

Zirconia is widely used due to its superior strength, biocompatibility, and aesthetic appearance. It has been extensively used in dental restorations such as crowns, bridges, dental implant, and implant superstructures. 1 2 However, the most common technical complication when using zirconia is the dislodgement of the restoration due to the small number of hydroxyl groups at the zirconia surface which were used to form the chemical bond to the resin cement. 3 4

The strong bond to zirconia is essential for long-term clinical success, but it is difficult to achieve because zirconia is chemically inert and non-etchable. Bonding to zirconia can be enhanced through both mechanical and chemical methods. 5 Mechanical methods, such as airborne particle abrasion with aluminum oxide (Al _2_ O _3_ ) or silicon dioxide (SiO _2_ ) particles, can improve bond strength. However, this method may induce zirconia grain transformation, contributing to the low-temperature degradation, which affected the mechanical properties of the material. 5 6 Chemical approaches offer an alternative to enhance the zirconia bond strength. 5 The use of acid-based primers, self-adhesive resin cements (SACs), or other resin-based cements can establish a durable bond by interacting with the hydroxyl groups on the zirconia surface. 7 8 Functional monomers in primers or cements, such as 10-methacryloyloxydecyl dihydrogen phosphate (10-MDP), 4-methacryloyl-oxyethyl trimellitate anhydride (4-META), and glycerol phosphate dimethacrylate (GPDM), play a crucial role in achieving the chemical adhesion. 9 10 However, the efficacy of these monomers to improve the bond strength varies due to the differences in their chemical compositions. 5 The previous study suggested treating the zirconia surface with both mechanical and chemical processes to increase the retention of the restoration. 11

Effective cementation plays a crucial role in establishing appropriate retention, marginal seal, and longevity of the restorations. The SAC, which contains acidic monomers, can be used without the need of etching or priming the tooth structure. It is widely used due to its excellent mechanical properties, low technical sensitivity, ease of handling, and biocompatibility. Despite conventional resin cement being reported to have higher bond strength than SAC, using an airborne particle abrasion combined with SAC can be considered adequate shear bond strength (SBS) clinically. 12 13

The thickness of the resin cement layer is a critical factor that affects the strength and durability of the adhesive bond between zirconia and the tooth structure. 14 A thick layer of cement may provide better stress distribution and reduce risk of fracture, but it may also decrease the bond strength due to the formation of voids, incomplete polymerization, or increasing the polymerization shrinkage. 15 16 Several studies have been conducted to evaluate the factor related to resin cement thickness, such as the design of the abutment preparation, the zirconia milling technique, the viscosity of resin cement, and the die spacer thickness. 17 18 Previous research found that an average occlusal space of 100 to 200 μm for ceramic milled on computer-aided design (CAD) and computer-aided manufacturing machines is common. However, it has been reported that an internal gap size of 50 to 100 μm is optimized for the performance of resin cement. 15 18 In 2019, a study by Taha and Ibraheem reported that using an 80 μm cement space with zirconia primer (Z-prime Plus) and SAC (RelyX Unicem Aplicap) achieved a significantly higher tensile bond strength than 100 or 120 μm layers. 19 Similarly, Maneenacarith et al found that using a 50 μm cement space with a combination of Clearfil Universal Bond, Clearfil DC Activator, and resin cement (Multilink N) provided the highest SBS among the tested groups. Notably, significant differences were observed between cement space thicknesses, with 240 μm yielding the lowest SBS. 20

Despite these findings, there is limited evidence on how cement thickness influences the bond strength of zirconia with different functional monomer-containing SACs when used without primer, as per the manufacturer's instructions. This study aims to evaluate the impact of resin cement thickness on the SBS of zirconia bonded with three different SACs. The null hypotheses are (1) the SBS between zirconia and SAC would not be significantly different across varying cement thicknesses and (2) the SBS between zirconia and SAC would not be significantly different across types of resin cement. The results of this study can provide valuable insights into the optimal cement thickness to achieve a strong and durable bond between zirconia and resin cement using SAC.

## Materials and Methods

### Specimen Preparation

One hundred and twenty specimens were used in this study. The sample size of this study was determined using mean SBS and standard deviations from the pilot study. A power analysis was performed to ensure the adequacy of the sample size using G*Power 3.1 for Windows (Heinrich Heine University Dusseldorf, Dusseldorf, Germany) with a significant level (α) of 0.05, a power of 0.95, and an effect size of 0.73. The result showed that the minimum sample size was 10 specimens per group. In summary, 120 specimens were required.

Dental zirconia specimens (A2 HT, Cercon, Dentsply Sirona, Bensheim, Germany), with the composition shown in Table 1 , measuring 8 mm in width and length and 6 mm in height were designed in a CAD software program (3D Builder, Microsoft, Washington, United States). The zirconia disk was milled in a milling machine (VHF K5 + , vhf camfacture AG, Ammerbuch, Germany) and sintered in the furnace (inLab Profire, Dentsply Sirona, North Carolina, United States) according to the manufacturer's instruction. The specimen blocks were measured to confirm the specimen's dimension using a digital vernier caliper (Digital Vernier Caliper, Mitutoyo, Kanagawa, Japan) and embedded in a polyvinyl chloride pipe using type IV dental stone (UltiRock, Whip Mix, Kentucky, United States), with only one side of the specimen exposed for bonding. The zirconia specimen was polished to a flat surface using 400- and 600-grit silicon carbide papers (3M Wetordry abrasive sheet, 3M-ESPE, Minnesota, United States), respectively, on a polishing machine (Nano 2000 grinder-polisher with FEMTO 1000 polishing head, Pace Technologies, Arizona, United States) with water running. The silicon carbide abrasive paper rotated counterclockwise at a speed of 250 rpm under a load of 2 kg/cm ^2^ for 1 minute. Surface of the zirconia specimen was treated by the airborne particle abrasion unit (Basic Quattro, Renfert, Germany), using 50 μm aluminum oxide particles, 10 mm away from the zirconia surface with a pressure of 2.5 bar for 15 seconds.

**Table 1:** The compositions of the materials used in this study

Trade names	Manufacturers	Compositions
Cercon HT, Shade A2 Lot number: 18047595	Dentsply Sirona, Bensheim, Germany.	Zirconium oxide, yttrium oxide 5%, hafnium oxide <3%, aluminum oxide, silicon oxide <1%
RelyX U200 Shade A2 Lot number: 9779427	3M-ESPE, Minnesota, United States	Base paste: methacrylate monomers containing phosphoric acid groups, methacrylate monomers, silanated fillers, initiator components, stabilizers, and rheological additives Catalyst paste: methacrylate monomers, alkaline (basic) fillers, silanated fillers, initiator components, stabilizers, rheological additives and pigments
PANAVIA SA LUTING Multi Shade universal A2 Lot number: 9T0208	Kuraray Noritake Dental Inc., Tokyo, Japan	Paste A: 10-methacryloyloxydecyl dihydrogen phosphate (10-MDP), bisphenol A diglycidylmethacrylate (Bis-GMA), triethyleneglycol dimethacrylate (TEGDMA), hydrophobic aromatic dimethacrylate, 2-hydroxymethacrylate (HEMA), silanated barium glass filler, silanated colloidal silica, dl-camphorquinone, peroxide, catalysts, pigments Paste B: hydrophobic aromatic dimethacrylate, silane coupling agent, silanated barium glass filler, aluminum oxide filler, surface treated sodium fluoride, dl-camphorquinone, accelerators, pigments
Maxcem Elite Chroma Shade white Lot number: 9197506	Kerr Corporation, California, United States	2-Hydroxyethyl methacrylate, 2-hydroxy-1,3-propanediyl bismethacrylate, 7,7,9 (or 7,9,9)-trimethyl-4,13-dioxo-3,14-dioxa-5,12-diazahexadecane-1,16-diyl bismethacrylate, propylidynetrimethanol, ethoxylated esters with acrylic acid, and ytterbium trifluoride
Filtek Z350 XT, Shade A2 Lot number: NCL2867	3M-ESPE, Minnesota, United States	Organic matrix: BisGMA, urethane dimethacrylate (UDMA), TEGDMA, and ethoxylated bisphenol-A dimethacrylate (BisEMA) Inorganic particle: nonagglomerated/nonaggregated zirconia, aggregated zirconia/silica

The zirconia specimens were randomly divided into 12 groups (10 specimens for each group) according to the 4 different thicknesses (50, 100, 150, and 200 μm) of 3 cement types (RelyX U200, 3M-ESPE, Minnesota, United States; PANAVIA SA LUTING Multi, Kuraray Noritake Dental Inc., Tokyo, Japan; Maxcem Elite Chroma, Kerr Corporation, California, United States).

The resin composite disks (Filtek Z350 XT Universal restorative, 3M-ESPE, Minnesota, United States) of 3 mm diameter and 2 mm height ( *n* = 120) were prepared in a putty silicone mold (Elite HD, Zhermack, Badia Polesine, Italy) and light-cured by a light curing unit (Bluephase N, Ivoclar-Vivadent, Schaan, Liechtenstein) intensity of 1,200 mW/cm ^2^ for 40 seconds in four different directions. Then, the specimens and resin composite disks were ultrasonically cleaned (VGT-1990 QTD, Guangdong GT Ultrasonic Co., Ltd, Guangdong, China) in distilled water for 10 minutes and air-dried.

### Bonding Procedures

The bond side of zirconia specimen was attached with an adhesive tape (Scotch 3M Tape, 3M-ESPE, Minnesota, United States). An abrasive tape was used to control the cement thickness; 50 μm = 1 layer, 100 μm = double layers, 150 μm = triple layers, and 200 μm = quadruple layers of an adhesive tape. The adhesive tape was punched into 2.38 mm in diameter to create the bond area.

**Fig. 1:**
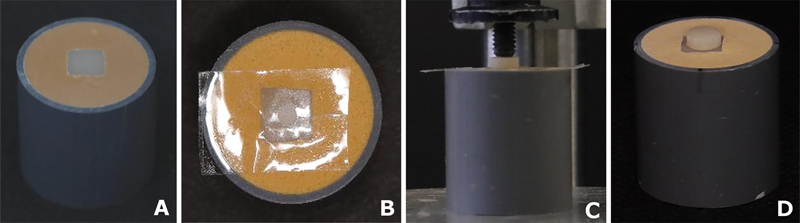
Sample preparation. ( **A** ) Zirconia specimen was embedded in the dental stone and polished to a flat surface. ( **B** ) The bond side of zirconia specimen was attached with a punched adhesive tape. ( **C** ) The resin composite block was bonded with the prepared zirconia specimen under a constant load using Durometer. ( **D** ) Zirconia bonded to the composite specimen.

The SACs used in this study are RelyX U200, PANAVIA SA LUTING Multi, and Maxcem Elite Chroma. The compositions of the cements are shown in Table 1 . The cements were prepared according to the manufacturer's instruction. Each cement was applied through an automix tip into the bond area according to the assigned group ( Fig. 1 ). The resin composite block was bonded with the prepared zirconia specimen under a constant load of 1,000 g with Durometer (ASTM D 2240 TYPE A, D PTC instruments, United States). The excess cement was removed by a micro-brush. Then, the bond surface was light-cured with an intensity of 1,200 mW/cm ^2^ for 20 seconds in four directions including the top surface of the specimen. After that, the adhesive tape was removed from the specimens. The cement thickness was initially checked by a stereo microscope (SZ61, Olympus Corporation, Tokyo, Japan) at 45× magnification.

### Incubation Process

All bonded specimens were immersed in distilled water and stored in the incubator (Contherm 160 M, Contherm Scientific Ltd., Wellington, New Zealand) at 37°C for 24 hours according to ISO 29022:2013. After 24 hours, the specimens were removed from the water and air-dried.

### Shear Bond Strength Test

The cement layer of the specimens was analyzed using a micro-computed tomography scanner (Micro-CT) with 1 mm aluminum filter (Skyscan 1273, Bruker Corporation; Massachusetts, United States). Tag Image File Format (TIFF) files were generated by a 90-kilovolt peak (kVp) with a voxel size of 11 μm and analyzed by an imaging processing software program (DATAVIEWER, Bruker Corporation, Massachusetts, United States). The software program was used to inspect slice-by-slice of three-dimensional volume before the test. The percentage of voids (V %) was calculated using the ImageJ program (Fiji 1.0, ImageJ, Wisconsin, United States).

The SBS of all specimens was tested using the universal testing machine (EZ-S 500N, Shimadzu Corporation, Kyoto, Japan) at a crosshead speed of 1.0 mm/min according to ISO 29022:2013. The notched-edge shear blade was placed parallel to the bond surface. The SBS (MPa) was calculated by dividing the maximum load at failure ( *N* ) with the bond area (mm ^2^ ) according to the following formula.

Shear bond strength = *F* (force)/ *A*_b_ (bond area).

### Mode of Failure Analysis

After the SBS test, specimens' surface was examined using a stereo microscope (SZ61 model, Olympus Corporation, Tokyo, Japan) to study the mode of failure at 40× magnification. The mode of failure was assessed by scoring the amount of the remaining cement on the zirconia surface. The area was calculated using the ImageJ program (Fiji 1.0, ImageJ, Wisconsin, United states). The criteria for scoring were adapted from the study conducted by Artun and Bergland. 21

Score 1 = All resin cement remains on the zirconia surface.
Score 2 = More than half of the resin cement remain on the zirconia surface.
Score 3 = Less than half of the resin cement remain on the zirconia surface.
Score 4 = No resin cement remains on the zirconia surface.

### Statistical Analysis

The Kolmogorov–Smirnov test at a significance level of 0.05 was used to evaluate the normality of the data. The data from the SBS test were statistically analyzed using two-way ANOVA (df: 6; *F* : 21.16; *p* < 0.05), followed by the Tukey HSD test to compare the differences in mean SBS at 95% confidence level (SPSS version 29 for Windows, SPSS, Chicago, Illinois, United States).

## Results

The Kolmogorov–Smirnov test indicated the normal distribution of each group ( *p* > 0.05). The mean SBS, standard deviation, and significant differences between groups are presented in Table 2 . Within the cement type, the group with the cement thickness of 50 μm exhibited the significant highest SBS and the 200 μm cement thickness group exhibited the significant lowest SBS ( *p* < 0.05). Considering the cement type, PANAVIA SA LUTING Multi exhibited the highest SBS.

**Table 2:** The mean shear bond strength and standard deviations of the groups (MPa)

Cement types	Cement thickness (μm)			
	50	100	150	200
RelyX U200	17.11 (± 1.97)	14.75 (± 1.53) ^a^	12.16 (± 1.15) ^c^	6.84 (± 1.09)
PANAVIA SA LUTING Multi	21.57 (± 1.39)	15.71 (± 0.94) ^a^	12.17 (± 1.57) ^c^	8.95 (± 1.27)
Maxcem Elite Chroma	13.16 (± 1.52)	9.96 (± 1.42) ^b^	9.51 (± 1.42) ^b^	5.51 (± 1.29)

Note: Same superscript letters represent no significant difference between groups ( *p* > 0.05) by two-way ANOVA followed by the Tukey HSD test.

**Fig. 2:**
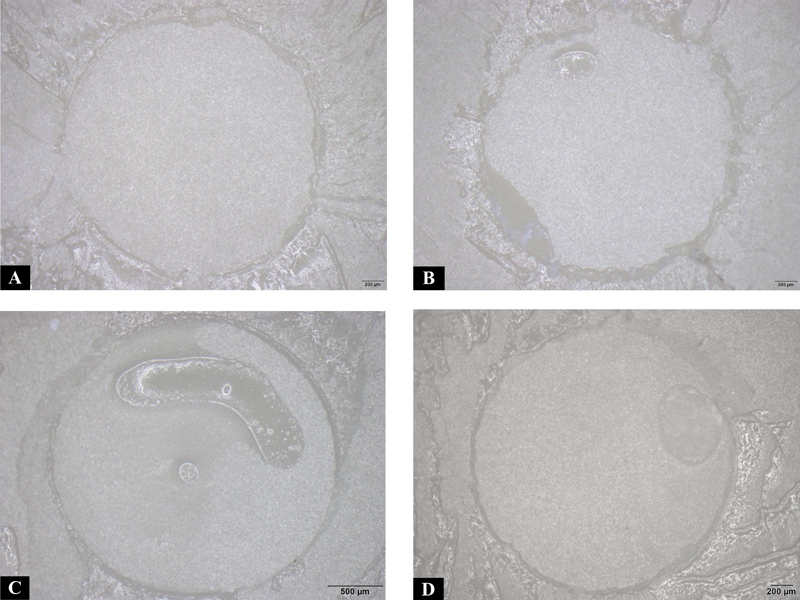
The morphology of the debonded zirconia surfaces using a stereo microscope at 40× magnification. ( **A** ) Debonded surfaces of zirconia using Rely X U200 with score 4. ( **B** ) Debonded surfaces of zirconia using Rely X U200 with score 3. ( **C** ) Debonded surfaces of zirconia using PANAVIA SA LUTING Multi with score 3. ( **D** ) Debonded surfaces of zirconia using Maxcem Elite Chroma with score 3.

Mode of failure is shown in Table 3 , with none of scores 1 and 2 were observed from the specimens. In PANAVIA SA LUTING Multi with the thickness of 50 μm groups, the failure was categorized as score 4. The predominant mode of failure in all groups, except for RelyX U200 and Maxcem Elite Chroma groups with a thickness of 50 μm groups, was score 4. In these exceptions, both score 3 and score 4 were equally represented ( Fig. 2 ).

**Table 3:** Mode of failure of the different groups

Cement type	Cement thickness (μm)	Mode of failure (% area)				
		Score 1	Score 2	Score 3	Score 4	Total
RelyX U200	50	0	0	10	90	100
	100	0	0	20	80	100
	150	0	0	40	60	100
	200	0	0	50	50	100
PANAVIA SA LUTING Multi	50	0	0	0	100	100
	100	0	0	20	80	100
	150	0	0	30	70	100
	200	0	0	40	60	100
Maxcem Elite Chroma	50	0	0	10	90	100
	100	0	0	30	70	100
	150	0	0	30	70	100
	200	0	0	50	50	100

**Fig. 3:**
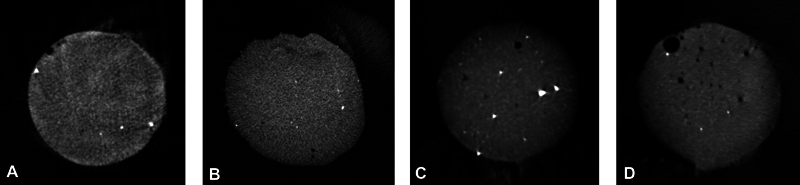
The Micro-CT images of the cement layer bonded with Maxcem Elite Chroma. ( **A** ) 50 μm cement thickness (0.34% void). ( **B** ) 100 μm cement thickness (0.38% void). ( **C** ) 150 μm cement thickness (0.79% void). ( **D** ) 200 μm cement thickness (1.79% void). CT, computed tomography.

According to the cement layer analysis, void was found in the cement layer images. Specimens with thicker cement layer showed more void than those of the thinner cement layer ( Fig. 3 ).

## Discussion

The purpose of this study is to evaluate the impact of cement thickness on the SBS of zirconia bonded with three different SACs. The effects of different thicknesses and types of cement were investigated. Considering the cement types, using a thickness of 50 μm demonstrated the significant highest SBS ( *p* < 0.05). The SBS decreased as the cement thickness increased. The SBSs of 50 and 200 μm cement thickness groups are significant differences across cement types ( *p* < 0.05). Therefore, the first null hypothesis, which is “the SBS between zirconia and SAC would not be significantly different across varying cement thicknesses,” was rejected.

Comparing the performance of different cement types, PANAVIA SA LUTING Multi exhibited the significant highest SBS compared with RelyX U200 and Maxcem Elite Chroma ( *p* < 0.05). Therefore, the second null hypothesis, which is “the SBS between zirconia and SAC would not be significantly different across types of the resin cement” was rejected.

To simulate clinical protocols for optimal zirconia bonding, all specimens were treated with aluminum oxide air abrasion and bonded with resin cement containing various acidic monomers. Air abrasion is recommended to generate surface roughness and increase the surface area, which enhances wettability, bond strength, and durability. 6 SAC was selected because it reduced technique sensitivity and ease of handling, making it more practical for clinical use. 13 The acidic monomers in the resin cement have an amphiphilic structure, allowing them to bond with both tooth structures and zirconia via functional groups, while polymerizing with the resin through their hydrolyzed groups. 7

As of now, no studies have specifically investigated the influence of resin cement thickness on the zirconia bonding using different SACs.

In this study, the 50 μm thickness was determined to be the minimum cement space that should be established. Gultekin et al indicated that in resin cements, increasing the cement space from 20 to 40 μm significantly improved retention. 21 22 However, the minimum cement thicknesses of 50 μm, which is the acceptable cement thickness of the International Standard Organization (ISO 4049:2019), was used in this study and the 200 μm thickness represents the maximum thickness.

The present study demonstrated that the 50 μm resin cement thickness, which was the thinnest cement layer used in this study, produced the significant highest SBS ( *p* < 0.05). These findings corresponded well with those of Taha and Ibraheem and Maneenacarith et al, who also observed a reduction in SBS as cement thickness increased. 19 20 In the present study, significant differences in SBS were found among the 50, 100, 150, and 200 μm cement thicknesses ( *p* < 0.05), except in Maxcem Elite Chroma between the cement thicknesses of 100 and 150 μm groups ( *p* ≥ 0.05).

A critical factor in the performance of SACs is their formulation, particularly the presence of specific acidic phosphate and/or carboxylate functional monomer. 13 In this study, the SBS varied across different resin cement thicknesses, PANAVIA SA LUTING Multi exhibiting the highest SBS, followed by RelyX U200 and Maxcem Elite Chroma, respectively. These differences in bond strength can likely be attributed to the distinct monomers found in each cement. 5 PANAVIA SA LUTING Multi contains 10-MDP, which possesses a hydrophobic long carbon chain, contributing to the superior hydrolytic stability. 23 10-MDP is a bifunctional adhesive monomer, which chemically interacts with the zirconium oxide layer on the zirconia surface via its hydrophilic phosphate terminal end and its hydrophobic methacrylate terminal end copolymerizes with the resin monomers in the cement. This chemical interaction is considered to be the primary reason for PANAVIA SA LUTING Multi to form the strong bond to zirconia. 23 24

RelyX U200, which contains a phosphate monomer derivative, demonstrated lower SBS compared with PANAVIA SA LUTING Multi. The phosphoric acid groups in RelyX U200 can form acid–base reactions with the hydroxyl groups present on zirconia, contributing to its bond strength, but not to the same extent as 10-MDP. 24 25 26 Maxcem Elite Chroma, containing GPDM, exhibited the weakest bond strength. GPDM's self-etching and adhesive properties may be effective for enamel and dentin, but its chemical adhesion to ceramic surfaces is less strong. 25 26 This weaker performance could be attributed to GPDM's higher hydrophilicity and shorter carbon chain, which likely reduces its interaction with the zirconium oxide layer. 26

Furthermore, the higher polymerization shrinkage stress associated with GPDM, due to its two polymerizable groups compared with the single group in 10-MDP, may contribute to its lower SBS. 26 This corresponds to the findings from Sriamporn et al and Fuentes et al, who suggested that the bond strength differences in SACs may be influenced by the acidic functional monomers. 25 26 The strong bonding ability of 10-MDP is likely due to its stable chemical interaction with zirconia, whereas the phosphoric acid derivative in RelyX U200 and the GPDM in Maxcem Elite Chroma offer weaker bonds, with GPDM exhibiting the lowest SBS due to its less favorable chemical characteristics. 24 25 26

The present study revealed a correlation between bond strength and failure mode. Groups with the higher SBS exhibited adhesive failures, whereas groups with the lower SBS showed a tendency toward mixed failures. Notably, only adhesive failures were observed in the group with the highest SBS (PANAVIA SA LUTING Multi with a thickness of 50 μm cement layer). The predominance of adhesive failure in zirconia–resin cement SBS tests can be attributed to the weaker bond at the zirconia–resin cement interface compared with the internal cohesive strength of the resin cement. 27

The images from cement analysis showed that the group with lower cement thickness, which exhibited high SBS, illustrated a lower percentage of voids in the cement layer ( Fig. 3 ). These findings align with the results of the finite element analysis conducted by May et al, which suggested that the higher SBS observed in the thinner cement layers is likely due to fewer voids and defects formed during the cementation process. 15 Polymerization shrinkage and incomplete polymerization within thicker cement layers can initiate crack propagation and weakening of the bond strength. 15 Moreover, these voids can act as stress concentrators when force is applied, potentially leading to crack initiation and increasing the failure transitioning from adhesive to mixed. 28

This study was an *in vitro* study and might not fully replicate the oral environment. Future research should investigate the impact of thermal stress on the durability of resin cement in the oral cavity. Moreover, the storage of specimens in water for 24 hours, following ISO 29022:2013, may not replicate the clinical environment. Utilizing thermocycling methods is suggested to simulate the thermal fluctuations experienced in clinical conditions. This study focused on only three different types of SACs, with one product selected to represent one type of the cement, other products should be evaluated in the future. In addition, investigations on the bubble-like defects observed on the remnant resin cement should be focused on to determine whether factors such as the method of cement application and the seating of specimens contribute to the formation of these defects.

## Conclusion

The difference in cement thickness and the type of SAC affected the SBS to zirconia. The lesser the cement thickness, the higher the SBS. 10-MDP-based cements which provide the highest bond strength to zirconia and a low cement thickness are suggested for the zirconia cementation. In clinical practice, the die space of 50 μm thickness is suggested for use in the zirconia crown CAD process. Moreover, preparation geometry, moisture control during crown fixation, and the other factors may affect the bond strength. Therefore, these factors are needed to be considered to achieve a high bond strength.
